# Biological consequences of zinc deficiency in the pathomechanisms of selected diseases

**DOI:** 10.1007/s00775-014-1139-0

**Published:** 2014-04-19

**Authors:** Kamil Jurowski, Bernadeta Szewczyk, Gabriel Nowak, Wojciech Piekoszewski

**Affiliations:** 1 Department of Analytical Chemistry, Faculty of Chemistry, Jagiellonian University, R. Ingardena 3, 30-060 Kraków, Poland; 2 Malopolska Centre for Translational Medicine, Faculty of Medicine, Jagiellonian University Collegium Medicum, Kraków, Poland; 3 Institute of Pharmacology, Polish Academy of Sciences, Kraków, Poland; 4 Faculty of Pharmacy, Jagiellonian University Collegium Medicum, Kraków, Poland; 5 Laboratory of High Resolution Mass Spectrometry, Regional Laboratory of Physicochemical Analysis and Structural Research, Faculty of Chemistry, Jagiellonian University, Kraków, Poland

**Keywords:** Zinc deficiency, Zinc diseases, Zinc pathomechanisms

## Abstract

From many points of view, zinc is one of the most important trace elements in biological systems. Many articles describe the well-known role of this metal in human physiology and pathophysiology, but in the related literature, there is a lack of current and reliable reviews of the role of zinc deficiency in many diseases. In this article, we describe the role of zinc deficiency in the oxidative stress control, immune response, proliferation, and pathogenesis and pathophysiology of selected diseases such as depression, cardiovascular diseases, diabetes mellitus, Alzheimer’s disease, and Wilson’s disease.

## Introduction

Is zinc deficiency a public health problem? There is no doubt that zinc is ubiquitous and one of the most important trace elements in biological systems. This metal plays an invaluable role in biological processes in different forms (e.g., zinc ions, zinc transporters, and the zinc albumin complex). Essentially, zinc in humans and its deficiency were first recognized in 1963, and since then, it has become apparent that zinc deficiency in humans is widely prevalent and significant. Zinc deficiency is widely prevalent throughout the world’s human population and has been observed in many countries. In this article, a current view of the role of zinc deficiency in the pathomechanisms of many diseases is presented.

## The physiological function of zinc

Zinc is ubiquitous and one of the most important trace element in biological systems. The exceptional ability of zinc atoms to participate in strong but readily exchangeable ligand binding, together with the notable flexibility in the coordination geometry of this metal, has proven to be extraordinarily useful in biological systems [1, 2]. Zinc performs its biochemical functions as a divalent cation primarily when bound to enzymes and other proteins. It is redox inert and has catalytic and regulatory roles in cellular biology [3]. This metal is indispensable to the growth and development of microorganisms, plants, and animals [3]. Zinc is essential as a catalytic, structural, and regulatory ion and is involved in homeostasis, immune responses, oxidative stress, apoptosis, and aging [4, 5].

Of all the trace element metals found in humans, only iron is more abundant than zinc. Hence, if hemoglobin-bound iron is not considered, zinc becomes the most abundant transition metal in the body [6–8].

This element can be found in all body tissues and secretions in relatively high concentrations with 85 % of all of the body zinc found in muscle and bones, 11 % in the skin and liver, and the remainder in other tissues with the highest concentrations in the prostate and parts of the eye [9]. The total zinc content of plasma is usually approximately 100 g zinc/100 mL of plasma depending on (1) age, (2) pregnancy, (3) sex, and (4) time of day as the plasma zinc content is higher in the morning than in the afternoon [10].

Zinc protecting biological structures from damage by free radicals may be due to several factors: an adequate level and maintenance of metallothioneins (MTs), an essential component of superoxide dismutase (SOD), a protective agent for thiols (RSH), thus preventing the interaction between chemical groups with iron to form free radicals [8].

Zinc is recognized as being important for stabilizing DNA and appears to reside in the nucleus longer than any other cell compartment. Therefore, it is possible that as intracellular levels of zinc increase, more iron will be displaced from nucleoproteins and less OH-driven DNA damage will occur. The underlying mechanism for the carcinogenicity of several heavy metals may be the dislocation of zinc from Zn-finger transcription factors and the release of oxygen radicals onto the DNA where they are bound. Zinc finger structures are potential targets for Cd(II) and Cu(II) [11].

Zinc has several specific functions in zinc enzymes: catalytic, co-catalytic (or coactive), and structural [10]. The catalytic role specifies that the metal is directly involved in enzyme catalysis. If zinc ions are removed by chelates or other agents, the enzymes lose their catalytic properties. The catalytic activity is eliminated because zinc itself is directly involved in the catalytic process. There is usually one catalytic zinc atom per enzyme subunit. Typically, the zinc atom is bound to three or four ligands, which comprise amino acids with histidine being the most frequent followed by glutamic acid, aspartic acid, and cysteine [10]. Water is the fourth and a universal ligand, but histidine is thus far the most frequent amino acid found in catalytic site residues. The regularity of amino acid spacing between the ligands of catalytic zinc atoms is characteristic [12]. Spacing between ligands has been classified as long and short. Long (5–196 amino acid residues) spacers stabilize structures and help align the residues that bind the substrate. Variations in the size and amino acid composition of long spacers could lead to the generation of structures that accommodate the binding of different substrates and could be involved in inducing a conformation essential to the enzymatic specificity. This long spacer arm could contribute to the induction of an active catalytic site, substrate-binding groups, and hydrogen bonds to form an active center [10]. The short spacer (1–3 amino acid residues) can form a bidentate zinc complex that could stabilize the overall and local protein structure by providing stiffness to the region affected, which is similar to disulfide bond formation in some proteins. Notably, short and long spacers that are characteristic of the catalytic sites of mono-zinc enzymes are repeated in co-active zinc metal bridges [12]. In most metalloenzymes, zinc plays a catalytic role, participating in the transformation of substrates by facilitating the formation of hydroxide ions at neutral pH or through Lewis acid catalysis. Zinc binds to many proteins in addition to metalloenzymes, particularly proteins involved in gene regulation [13].

Additional zinc or other metal sites have been termed co-catalytic (or coactive). This dependence was established 50 years ago. Zinc has a critical association with the enzyme carbonic anhydrase [14]. Co-catalytic (coactive) zinc atoms increase or decrease catalytic function in conjunction with another active site zinc atom in the same enzyme, but are not indispensable of itself for either enzyme activity or stability [10].

A characteristic zinc site form exists in zinc enzymes that contain two or more metal atoms that function as a catalytic unit. Co-catalytic zinc-binding sites are present in enzymes that contain two or more zinc atoms in close proximity to one another. Amino acids form ligand bridges between two zinc atoms or a zinc atom plus a different metal [12]. Structural zinc atoms are necessary only for the structural stability of the protein and can help stabilize the quaternary structure of oligomeric holoenzymes. Zinc plays a structural role in enzymes such as alcohol dehydrogenase, aspartate transcarbamylase, and protein kinase C [10].

## Zinc homeostasis: absorption, transport, and excretion

It is known that the basic zinc homeostasis maintenance mechanism is related to changes in zinc absorption and secretion to and from the alimentary tract. It is associated also with regulation of zinc urinary excretion and its tissue and cell redistribution [15].

There are two absorption possibilities for this metal depending on several factors, including the amount of zinc, the type of food consumed, and the body zinc requirements [16]. If zinc intake is low, absorption takes place mainly by a carrier-mediated process [8]. It is possible that the exocrine pancreas secrete a ligand (of which metallothionein is a candidate) that enhances jejunal zinc absorption. If the ligand is not saturated, it binds dietary zinc in the lumen of the intestines and facilitates its absorption. The zinc in the intestinal lumen comes from two sources: the diet (~10 mg) and digestive juices (~3 mg). The highest concentration of zinc contains pancreatic juice and may contain a ligand that promotes the absorption of zinc in the intestine [17]. It can be generally assumed that an intraluminal transition occurs to allow zinc to be transported across enterocytes as free ions [8]. The presence of phytic acid, calcium and trace metals such as cadmium, mercury or copper retards its uptake, but the presence of glucose in the intestinal lumen assists its uptake. Absorption most likely takes place by passive diffusion and carrier-mediated processes with zinc entry increasing in relation to requirements. Information about the absorption of zinc through the skin is limited [10, 16], and some absorption of zinc through damaged skin does occur in burn patients treated with zinc oxide dressings [18]. A decrease in zinc absorption is associated with age, but zinc excretion also decreases with age. Therefore, the overall zinc homeostasis is not deregulated with age [17].

**Fig. 1:**
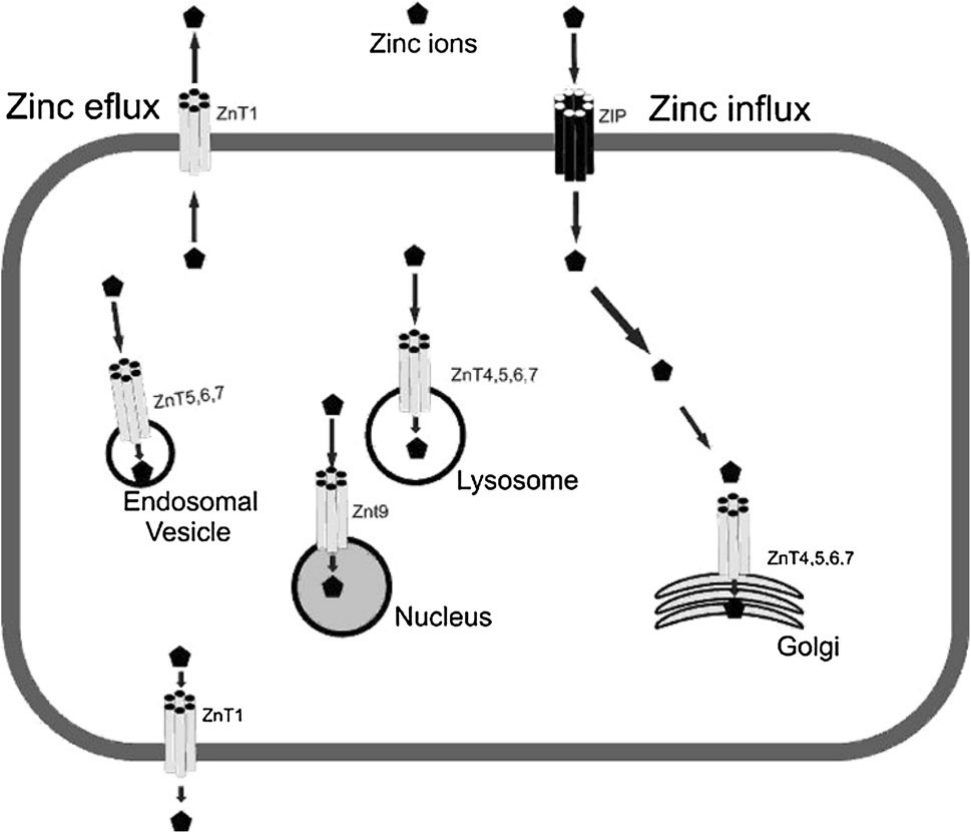
Possible role of zinc transporters in cells

A zinc transport mechanism in intestinal epithelial cells remains a subject of research and discussion. In the brush-border membrane of the proximal small intestines of rats, divalent metal-ion transporter-1 (DMT1) has been identified to have substrate specificity for the following cations: Zn^2+^, Fe^2+^, Mn^2+^, Cu^2+^, Cd^2+^, Ni^2+^, Co^2+^ and Pb^2+^ [17, 19]. In intestinal epithelial cells, zinc binds to metallothioneins or other intracellular proteins, such as cysteine-rich intestinal proteins, and is also transported into organelles and across the basolateral membrane into blood. One of the intracellular protein zinc transporters, ZnT-1, has been localized to the basolateral membrane of the intestinal epithelial cells of rats, protecting cells from metal toxicity, and is a likely candidate for zinc export from cells. ZnT-2 is another zinc transporter and has been localized to intracellular vesicles in rat kidney cells [8]. This transporter (ZnT-2) may play an important role in transporting zinc into vesicles in addition to protecting cells from zinc toxicity. Zinc is exported to the mesenteric circulation from the basolateral membrane of intestinal epithelial cells where it binds to plasma proteins (mainly albumin) [17]. Protein and zinc adducts are then transported to the liver via the portal circulation where it is absorbed and released and then distributed to other tissues. In the serum, zinc is bound primarily to albumin (85 %), α_2_-globulins (16 %) [16], and amino acids (1–2 %) [17] It is well known that a major zinc transporter is Zip5 (Slc39a5), which regulates intestinal zinc excretion and protects the pancreas against zinc toxicity. Figure 1 summarizes the roles described for Zn transporters.

## The role and distribution of zinc in the bran

There is a lack of storage of zinc in the body [17]. Endogenous zinc can be excreted via different ways, including the intestines, kidneys, integument, and semen [20]. The major route of zinc excretion is the intestine. Small amounts of zinc are lost in urine, desquamated skin, hair outgrowth, semen, and menses. Unabsorbed zinc (endogenous and dietary) and zinc present in sloughed epithelial cells are excreted from the body via stool. The amount of zinc lost in the stool is connected with diet: 1 mg/day for a low-zinc diet and 5 mg/day for a high-zinc diet. With regard to the high concentration, zinc is excreted via urine. Large losses of zinc by urine are associated with the nitrogen that is lost via this manner, which in turn is associated with stress, infection, burns, major surgery, or trauma. One explanation for this phenomenon could be protein catabolism [17, 20].

When compared with all organs, the brain presumably contains the highest levels of zinc in the body with a possible exception of pancreatic β islets [21]. The highest level of this element in the brain is found in the gray matter of the forebrain, reaching 60–90 ppm. The white matter has the second highest level as it contains slightly lower levels of zinc (26–40 ppm), which may be due to the lower water content located here [21]. An interesting fact is that a significant fraction of zinc in the brain (10–15 %) is localized in the synaptic vesicles of certain glutamatergic neuron terminals [22].

Approximately 80 % of total brain zinc exists as zinc metalloproteins, while the rest mainly exists in presynaptic vesicles and is histochemically reactive as revealed by Timm’s sulfide-silver staining method [23]. Modified Timm’s staining and staining with a zinc-selective fluorescent quinoline derivative, *N*-(6-methoxy-8-quinolyl)-para-toluenesulfonamide (TSQ), confirm a willingly accessible zinc entity in much of the neuropil throughout the telencephalon. “Vivid” zinc staining is present in neocortical layers, i.e., I–III and V, the hippocampus (dentate gyrus, CA1-4 radiatum, and oriens and mossy fibers), subiculum, amygdala, thalamus, and striatum, while much less staining is found in the cerebellum, brain stem, and spinal cord [14].

High levels of synaptic zinc are mainly present in the limbic systems, including the mossy-fiber terminals of hippocampal dentate granule neurons with a total zinc concentration of 136–145 ppm. Other brain regions, such as the thalamic dorsomedial, reticular nuclei, and neocortical layers II–III and V, are also enriched with zinc-containing terminals. However, the brain stem, cerebellum, and spinal cord are low in zinc-containing terminals [21].

**Fig. 2:**
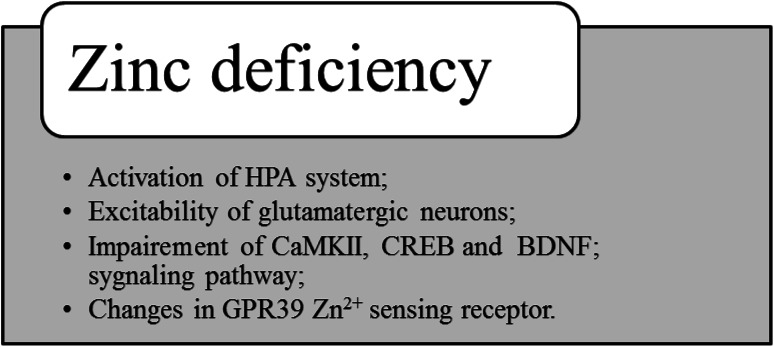
Possible mechanisms involved in the behavioral changes and brain functions observed in zinc deficiency

Possible mechanisms involved in the behavioral changes and brain functions observed with zinc deficiency are summarized in Fig. 2.

## Deficiency of zinc and organism malfunction

Zinc deficiency is the most significant pathological state involving metal metabolism abnormalities in the body [24, 25]. The risk of zinc deficiency affects approximately 50 % of the world’s population [25]. This state in humans occurs in populations whose diets contain a powerful chelator, high phytate concentration (cereal-based diets), and low protein because these factors result in the binding of biogenic zinc [8, 17, 26]. Zinc deficiency in developing countries affects nearly two billion people, mainly because of the high level of phytate in their diet (beans and bread), which impairs the absorption of this element [8]. The minimum zinc requirements in humans corresponding to health, acceptable growth, and well-being change with climate conditions, the existence of stress imposed by trauma, the type of diet consumed, and parasitic infestations and infections. The commonly recommended daily dietary Zn requirement is 15 mg/day [8], and the tolerable upper intake level of this metal is recommended to be 25 mg/day [27].

Organ systems that are known as being clinically affected by severe zinc deficiency include the central nervous, gastrointestinal, immune, epidermal, reproductive, and skeletal systems [1], which is due to increased requirements or excretion, inadequate dietary intake, conditioned deficiency, or genetic causes [10].

Zinc deficiency results in the death of children with the zinc malabsorption syndrome acrodermatitis enteropathica because of infections caused by decreased lymphocyte counts due to atrophy of the thymus size and function [28]. Acrodermatitis enteropathica is a hereditary zinc deficiency disease manifested by baldness, ulcerated and hypertrophic skin, muscle wasting, and chronic diarrhea [16]. This condition is a rare inherited autosomal recessive disease caused by decreased intestinal zinc absorption. The gene responsible for this disorder is SLC39A4 [29, 30]. For this disease, the plasma zinc and alkaline phosphatase levels are characteristically low [17]. A better understanding of the disrupted biology underlying each of the clinical features of acrodermatitis enteropathica primarily includes determining the importance of the optimal medical management of patients with this autosomal recessive inherited disease and other severe zinc deficiency disorders [13, 31]. Zinc deficiency has been described both in animals and humans after a prolonged reduction in intake or excessive uncompensated loss. This essential metal plays an important role in protecting cells from oxidative stress [32]. Long-term deprivation of this trace element makes an organism more susceptible to damage induced by oxidative stress. More specifically, zinc deficiency increases the osmotic fragility of erythrocyte membranes and the levels of lipid peroxidation in mitochondrial and microsomal membranes, while the presence of zinc prevents lipid peroxidation [10].

Insufficient zinc absorption causes an immediate decrease in protein turnover and cell growth to preserve zinc body pools, which can lead to rapid signs of zinc deficiency [17].

Nutrient malabsorption can be made by a variety of diseases (extraintestinal and intestinal), dietary products, drugs, and procedures (such as intestinal resection or bypass surgery). Diseases that most commonly cause zinc deficiency include short-bowel syndrome, celiac disease, Crohn’s disease, HIV, and enterocutaneous fistulae. Zinc deficiency can also cause pancreatic insufficiency, but the etiology is poorly understood [17, 33, 34].

Hypogonadal dwarf syndrome is connected with zinc deficiency characterized by hypogonadism and growth retardation along with dysosmia, anemia, dysgeusia, poor wound healing, and HSM (hepatosplenomegaly) [16]. Zinc deficiency is also connected with diarrheal diseases. This correlation has been best studied in children. Zinc supplementation lessens the duration and frequency of diarrheal episodes in underfed children. Children with malnutrition have the highest mortality and morbidity rates and are more likely to have long term and more rife episodes of these diseases. Children are also more likely to be zinc deficient due to an initial decrease in dietary intake followed by increased losses via stool [17]. An abnormally low zinc concentration in breast milk has been associated with the development of zinc deficiency in breast-fed newborns characterized by acrodermatitis, irritability, and delayed growth. Albumin-bound zinc in plasma is the metabolically active pool of zinc in the body. Zinc in bone is relatively inert except during periods of calcium mobilization from bone [16]. In contrast, epidemiological studies have associated low plasma zinc levels with abnormal pregnancy outcomes, and controlled intervention trials showed that zinc repletion improves pregnancy outcome [35].

## Deficiency of zinc in depression

Depression is a psychiatric disorder characterized by high morbidity and mortality (suicide) [36, 37]. There is substantial documentation correlating zinc deficiency and depression. However, the pathophysiology of depression via zinc deficiency remains poorly understood.

The general hypothesis concerning the lack of zinc in relation to a depressive disorder was first suggested in the 1990s when McLoughlin and Hodge [38], Maes et al. [39], and Nowak [40] found significantly lower serum zinc levels in patients with depression than that observed in healthy controls. Subsequent studies confirmed this hypothesis by showing a low zinc level in different types of depression. Moreover, it was shown that the zinc concentration might differ depending on comorbid disorders, patient status, or severity of depression [41–45]. However, there are only a few clinical studies directly indicating the relationship between dietary zinc intake and depressive symptoms. A study performed by Amani et al. [46] reported decreased daily zinc intake and decreased serum zinc concentrations in young depressed women as compared to healthy volunteers. Another study showed that lower zinc intake, higher stress and social disadvantage were associated with the occurrence of depressive symptoms and that this effect was attenuated by higher zinc intake [47].

A growing body of evidence demonstrates that experimentally obtained zinc deficiency induces depressive-like behavior and has suggested this procedure as a new animal model for depression. Experimentally, zinc deficiency is achieved by a diet that contains 0.5–6 mg Zn/kg for at least 2–4 weeks [48, 49]. Animal studies showed that dietary zinc deprivation increases the immobility time in the forced swim test and tail suspension test in mice and rats, thus indicating pro-depressive behavior [48–51]. Furthermore, zinc deficiency reduces the consumption of sucrose in rats, which indicates the appearance of anhedonia (one of the common symptoms observed in human depression) [51]. Rats fed with a zinc-deficient diet also exhibit anorexia and anxiety-like behavior [51]. Other data have indicated that low zinc intake impairs zinc homeostasis in the brain, decreases the level of synaptic zinc, and interferes with the processes of learning and memory. It is interesting to note that a zinc-deficient diet given to young adult rats produced an impairment in learning and memory, and this could be reversed by feeding with a normal diet [52], while such impairments caused by diet administration during development are irreversible [52, 53]. Other effects reported to be induced by zinc deficiency include a decrease in the number of progenitor cells and immature neurons in the rodent hippocampus, indicating disrupted neurogenesis and synaptogenesis [55–58].

One possible mechanism involved in the behavioral changes and brain functions observed in zinc deficiency is activation of the hypothalamo-pituitary-adrenocortical (HPA) system. Indeed, an increased corticosterone concentration was found in young rats and mice fed with a zinc-deficient diet [49, 54]. The other possible mechanism responsible for zinc deficiency-induced effects is the excitability of glutamatergic neurons [49]. The best example of how zinc modulates glutamatergic neurotransmission is the inhibition of the glutamate *N*-methyl-d-aspartate (NMDA) receptor [59]. In zinc deficiency, this inhibition activity declines and results in increased glutamate release, prolonged intracellular Ca^2+^ elevation and excitotoxic damage (see [60]). Another way in which zinc deficiency can disrupt brain function is impairment of CaMKII (Ca^2+^/calmodulin-dependent protein kinase II) and CREB (cAMP-response element-binding protein) signaling pathway (see [61]).

The other biochemical effects induced by zinc deficiency that may be involved in the pathomechanism of depression include a reduction in the protein level of brain-derived neurotrophic factor (BDNF), tropomyosin-related kinase B (TrkB) receptor and GPR39 Zn^2+^-sensing receptor [62].

## Deficiency of zinc in cardiovascular and renal diseases

Zinc plays a role in gene expression and is crucial for processes associated with cells, such as differentiation, cell division and protein synthesis, and this metal is also important in the development of many organs, e.g., the heart and kidneys [4, 61]. It is well known that zinc supplementation to normal healthy and elderly subjects (for 6 months) lowers oxidative stress markers and the production of C-reactive protein and inflammatory cytokines in macrophages and monocytes [64]. In turn, the consequences of zinc deficiency include (1) generation of apoptosis, (2) oxidative stress, and (3) inflammation processes in tissues that can contribute to the development and/or maintenance of several cardiovascular diseases (arterial hypertension, atherosclerosis, congestive heart failure and coronary heart disease) and renal insufficiency [65].

Many researchers have reported a correlation between the changes in zinc metabolism that lead to zinc deficiency and the etiopathogenesis of cardiovascular diseases such as primary arterial hypertension [66, 67]. Studies regarding the role of zinc in the regulation of blood pressure and the pathogenesis of arterial hypertension began after the observation that there is an inverse correlation between the level of arterial pressure and serum zinc concentration [68]. It is known that the changes in the initial stage of hypertension disease and zinc metabolism are connected via the activity of Renin–angiotensin–aldosterone system (RAAS) arrangement, and the primary change causing the others is the increase in zinc excretion in urine [67]. A study comparing the concentration of zinc in the serum of rats with spontaneous arterial and renal hypertension and those that are normotensive found that it was higher in rats with spontaneous arterial hypertension compared with the other groups. Serum zinc in normotensive rats and rats with renal hypertension did not differ [69]. Moreover, outflow constants for zinc from lymphocytes in patients with mild arterial hypertension were higher than in a group of healthy people although the outflow constants for zinc from the lymphocytes of patients with moderate arterial hypertension did not significantly differ in comparison with a group of healthy people [67]. During the process of arterial hypertension, changes in zinc metabolism most likely result from a deficit in this trace element as the illness persists. It is clear, therefore, that zinc has great importance in the regulation of arterial pressure and the etiopathogenesis of arterial hypertension. Nonetheless, it is difficult to evaluate the importance of changes primarily leading to the development of hypertension.

It is known that heart development can be particularly sensitive to zinc deficiency. Moreover, the heart is also an important target of the fetal programming of cardiovascular diseases in adult life. In rat experiments, severe maternal zinc deficiency has been associated with a high incidence of fetal heart anomalies, which were speculated to result in part from the reduced expression of heart-specific genes that contain zinc-finger transcription factor binding sites in their promoter sequence [70, 71].

Atherosclerosis is characterized by the slow development and subsequent rupture of vulnerable plaques leading to heart attacks and strokes with sudden death or later death from heart failure [72]. The role of zinc metabolism in atherosclerosis is well known. First, zinc cations are rapidly taken up by endothelial cells (possibly by the endocytosis of albumin-bound zinc). Albumin-bound zinc is the largest pool of bound zinc in plasma, which also binds to large macromolecules such as α2-macroglobulin. The plasma protein-bound zinc pool quickly turns over in rapid equilibrium with total plasma zinc; hence, changes in dietary zinc (including deficiencies) have the potential to alter the endothelial cell levels of zinc. It is known that endothelial cells undergo apoptosis possibly as a result of increased oxidative stress from long-chain fatty acids, oxidized LDL, or inflammatory cytokines derived from activated monocytes. There is evidence that zinc may play a protective role in maintaining the integrity of endothelial cells [73]. As such, there is decreased vessel susceptibility to atherosclerosis. It follows that corollary zinc deficiency may promote endothelial cell injury. A mediator of inflammatory responses in many cells is the transcription factor nuclear factor-kB (NF-κB), which regulates gene expression associated with apoptosis and inflammation. The process of NF-κB binding to DNA is dependent on zinc; therefore, NF-κB transcriptional activity is regulated by this metal. In endothelial cells, the zinc ionophore and pyrithione inhibit the regulatory activities of NF-κB [74]. A step of major importance in the development of atherosclerosis is the upregulation of adhesion molecule expression on endothelial cells, a process mediated by NF-κB. Accordingly, it is likely that zinc can regulate this critical step in atherosclerosis in an inhibitory manner when the zinc status is high and in a proatherogenic manner in states of zinc deficiency [75].

Heart failure is a clinical syndrome that includes reduced cardiac output, tissue hypoperfusion, tissue congestion, and increased pulmonary capillary wedge pressure. Many enzymes essential for adequate function of the cardiovascular system are zinc dependent [76]. It is well known that the serum zinc level is significantly lower in children with congestive heart failure [77]. This disease may be associated with zinc deficiency via reduced dietary intake, reduced absorption due to gastrointestinal edema and impaired motility, increased intestinal zinc loss, and excessive urinary excretion due to the use of diuretics. Furthermore, the majority of heart failure patients are older, and many of them have various co-occurrences, which may further impair zinc metabolism [76].

The preservation of renal function is connected with zinc. Based on laboratory tests in rats, it was found that severe zinc deficiency in adult rats induces a decrease in the glomerular filtration rate and renal blood flow, and increases renal vascular resistance. It appears that these changes are associated with enhanced superoxide anion formation through low Cu/Zn SOD activity in the kidneys of zinc-deficient rats [78]. Zinc deficiency may also play a role in the progression of renal failure. A predominant effect of renal insufficiency on zinc homeostasis is hypozincemia, which occurs due to increased urinary zinc excretion [65].

## Deficiency of zinc in diabetes mellitus

Diabetes mellitus is a group of disorders characterized by hyperglycemia and can be clinically classified as insulin dependent (type I diabetes mellitus, IDDM) or non-insulin dependent (type II diabetes mellitus, NIDDM) [79, 80]. Metabolism in diabetes is characterized by an abnormally high concentration of blood glucose [81]. This condition is because glucose is the major stimulus of insulin secretion in human beings. This process is tightly regulated by the following: (1) the coordinated action of nutrients, (2) gastrointestinal and pancreatic hormones, and (3) autonomic neurotransmitters. In humans and animals, diabetes causes disturbances in zinc metabolism. Zinc is necessary for insulin synthesis and storage [82, 83]. Insulin exists in hexameric crystals containing a variable number of zinc atoms that are stored in β-cells and released into the portal venous system at the time of the β-cells’ degranulation [81, 82, 84]. In pancreatic β cells, a novel member of the ZnT family gene, SLC30A8 (the gene of ZnT8), was identified a decade ago. ZnT8 is specifically expressed and has been identified as a novel target autoantigen in patients with type 1 diabetes [85]. In type 2 diabetes, a single nucleotide polymorphism in SLC30A8, rs13266634 (Arg325Trp), has been reported. The ZnT8 transporter is located on dense core vesicles (DCVs) in β cells and loads the zinc cation into these secretory compartments where it then binds with and stabilizes the hexameric form of insulin [89]. It appears that functional ZnT8 facilitates autocrine and paracrine roles for the zinc ion bursts produced by β cells upon glucose stimulation [86]. Insulin is secreted as zinc crystals. Zinc ions and insulin create a hexameric, crystalline structure, comprising two Zn^2+^ ions and six molecules of insulin, which is stored in secretory granules until secretion in response to metabolic demands. Therefore, zinc plays an important role in modulating the immune system, and its dysfunction in diabetes mellitus may be connected to the status of this metal [87, 88]. It is known that a part of the zinc cation pool in the β cell is co-secreted with insulin after stimulation with glucose. After secretion, the hexameric structure of insulin dissociates into the active monomer and zinc ions, which is most likely caused by a combination of a rapid decrease in Zn^2+^ pressure. Moreover, an in vitro study suggested that the Zn ions co-secreted with insulin during hyperglycemia might contribute to β cell death via a paracrine mechanism [89]. It has been hypothesized that such activity could link hyperinsulinism with β cell necrosis and the ensuing type 2 diabetes. The indispensable role of zinc in the insulin structure makes it a requirement when insulin analogs are prepared to ensure its activity and stabilization [89]. It is well known that decreased levels of this metal are observed in diabetes mellitus. Moreover, in both type I and type II diabetic individuals, hyperzincuria and indications of zinc malabsorption have also been observed. Perhaps zinc deficiency in diabetics could result from hyperglycemia or impaired intestinal zinc absorption, but in the absence of compensatory mechanisms, excessive zinc loss in urine may be the main cause of inducing a deficient or marginal zinc status [90]. Alternatively, the effect of zinc deficiency on insulin secretion by the pancreas could be one of the mechanisms of glucose intolerance. Zinc also plays a role in insulin behavior because it can enhance insulin binding to hepatocyte membranes [91]. In studies of isolated adipocytes from rats, it was found that there is no impaired insulin binding in zinc-deficient and pair-fed rats. This observation suggests that caloric restriction is secondary to zinc deficiency and plays a role in insulin resistance, which was confirmed by in vivo studies [92]. Because glucose is the major stimulus for insulin secretion and this process is tightly regulated by the coordinated action of zinc, the effects of the deficiency of this metal on peripheral glucose metabolism could be related to the action of zinc on glucose transporter translocation inside cells or modification of the glucose transporter structure. Many studies indicate that Zn deficiency significantly decreases the response of tissue to insulin [84].

Zinc has insulin-like effects on cells such as the promotion of lipogenesis and glucose transport. Hence, zinc supplementation to diabetic patients may stimulate tissues to use glucose and maintain normal cellular function and normal lipid metabolism. It is known that supplementation of this element enhances the gastrocnemius insulin receptor concentration and tyrosine kinase activity. In zinc-treated diabetics compared the non-zinc-treated diabetics, the fasting serum glucose concentration was significantly lower, and there was a negative correlation between femur Zn and the serum glucose concentration [93]. Moreover, supplementation of this element markedly ameliorated the hyperglycemia of diabetic mice together with an increase in leptin production, and it appears that zinc is a mediator of leptin production [94]. In contrast, zinc acts on insulin function through a direct effect on insulin signaling and indirect action on insulin-like growth factor (IGF) regulation [95–97]. Stimulating multiple components, such as tyrosine phosphorylation of the insulin receptor β subunit, phosphoinositide (PI) 3-kinase, tyrosine phosphorylation of insulin receptor substrate (IRS)-1, and serine-473 phosphorylation of Akt, is associated with the effects of zinc on insulin signaling [98, 99].

Oxidative stress plays a critical role in the development of diabetic complications. Thus, diabetes prevention suppressing systemic antioxidant capacity may be a reason for the prevention of systemic diabetic complications by zinc supplementation [100]. One of these mechanisms explains the role of zinc as a biological antioxidant. In fact, some research has shown that lipid peroxidation increases during diabetes mellitus, and superoxide dismutase (SOD) shows low activity [100]. Accordingly, this increased peroxidation could affect insulin or glucose transporter function by having a direct effect on proteins or an indirect effect on membrane fluidity. Moreover, it is known that zinc protects cells from oxidation damage by free radicals due to the essential SOD [5, 101].

The beneficial effects of zinc supplementation in diabetes mellitus type-1 and type-2 were observed in animal (fasting insulin level and fasting glucose in mice) [82] and in human studies [102, 103]. However, in type-1 diabetes, studies have reported a negative effect of zinc supplementation on glucose homeostasis [104].

## Deficiency of zinc in Alzheimer’s disease (AD)

Alzheimer’s disease was first described by Alzheimer [105]. This disease is reflected in elderly people and usually begins with memory loss, known as mild cognitive impairment (MCI). At least 80 % of patients with MCI develop full-blown AD at a rate of approximately 15 % per year. The main hypothesis for the pathogenesis of AD is the amyloid hypothesis because amyloid plaque formation is so intimately connected to AD [106, 107]. The main point of this hypothesis is that there is evidence that oxidant damage occurs in the brains of patients with AD and neurofibrillary tangles, and amyloid plaques generate toxic oxidant radicals. Oxidant radicals are produced particularly by the presence of excess copper or iron [108, 109]. It is known that the risk factors for developing AD include (1) age [105], (2) having the E4 allele of apolipoprotein E [110], (3) elevated homocysteine level, (4) having certain hemochromatosis [111] and transferrin alleles [112], and (5) fat in the diet [113]. It is also well known that the Zinc serum concentration decreases with aging and indeed, patients with AD decline more rapidly, thus making them zinc deficient when compared with age-matched controls [114, 115]. ZnT3 mediates age-related AD-like β-amyloid (Aβ) neuropathology in transgenic mice [116, 117]. This zinc transporter (Slc30a3 gene) is essential for loading zinc ions into synaptic vesicles [118]. ZnT3 is mainly localized to the glutamatergic synapses [119] present in regions of the brain, such as the hippocampus and neocortex, mediating higher cognitive functions. This transporter most likely plays a main role in the modulation of synaptic transmission and plasticity because ZnT3 likely represents a major (probably sole) synaptic vesicular zinc ion transporter, and may regulate the downstream effects of synaptic Zn^2+^ in a variety of signaling pathways [120]. Furthermore, the ZnT3 transporter level decreases with aging in the brains of mice and humans, and decreases even further with aging in the brains of patients with AD when compared with age-matched controls. Additionally, the extracellular amyloid plaques in AD brains are willing zinc binders, further depleting available zinc for neurons [120, 121]. Alternatively, another mechanism involved in AD and zinc action can be zinc’s capacity to inhibit calcineurin [122]. It has been postulated that a causative factor for AD is increased neuronal calcineurin activity because it adversely affects many downstream biochemical functions. Calcineurin activity is increased by exposure to β-amyloid and inhibited by zinc. It appears that neuronal zinc deficiency plays an important role in decreasing neuronal function and increasing damage, leading to cognition loss in Alzheimer’s disease. It is possible that excess copper and its toxicity leads to amyloid plaque development, and the plagues then trap increasing amounts of Zn [123]. This zinc decrease effect is connected with the zinc depletion and the loss of ZnT3 function with age, which is exaggerated in Alzheimer’s disease and leads to severe neuronal zinc deficiency and neuronal damage. Hence, it is highly probable that zinc therapy might be helpful for Alzheimer’s disease. The first research performed on this subject involved an attempt at therapy (orally and parenterally) in patients with AD in 1992 by Constantinidis [124]. This study reported that there was a substantial improvement in cognition, but this was an uncontrolled study. The latest study of zinc therapy in a mouse model of Alzheimer’s disease likewise reported improved cognitive performance when compared with placebo controls [125]. It can be concluded that zinc plays an integral role in the mechanism of Alzheimer’s disease with high probability, thus supporting the hypothesis that restoring zinc homeostasis might be beneficial for the treatment of AD [61].

## Deficiency of zinc in Wilson’s disease (WD)

Wilson disease is an autosomal, recessively inherited, inborn error involving abnormal copper handling by the liver [126]. WD is caused by mutations in the ATP7B gene, which leads to copper accumulation in various organs and predominantly presents hepatic or neuropsychiatric symptoms [127]. These symptoms are characterized by impaired hepatocellular utilization and biliary copper excretion. This condition leads to copper accumulation in the liver, brain, cornea, kidney, and other organs, eventually causing end-stage liver disease and severe brain damage [128]. The main objective of medical treatment is the reduction of accumulated copper in tissues and maintenance of an adequate [2] concentration of this metal in the body. There is a lack of clinical trials comparing different therapeutic strategies, which presents a serious disadvantage for Wilson disease patients. It is possible to use three drugs for the treatment of WD: (1) d-penicillamine, (2) trientine, and (3) zinc salts [127, 128]. The mechanism for all of these drugs is based on chelation. Symptomatic patients have an extensive copper overload, and the primary purpose of pharmacological treatment is a negative copper balance. Chelation therapy with d-penicillamine or trientine is given until free serum copper and urinary copper excretion is within the normal range. In most examples, this therapy lasts from 6 to 12 months or even longer [129]. However, a patient with Wilson’s disease treated with penicillamine can also have zinc deficiency. This zinc deficiency presumably occurs as a result of the chelating effects of penicillamine, which depletes the body of its zinc content [26, 28].

To maintain the copper and zinc levels in balance after previous copper reduction, maintenance therapy is required. Thus, low-dose chelators or zinc salts are used. In this case, zinc salts block the intestinal absorption of dietary copper by stimulating the synthesis of the endogenous copper chelator metallothionein. In turn, metallothionein-bound copper is excreted by the desquamation of enterocytes within several days via feces [130]. Zinc salts are highly suitable for therapy during the presymptomatic stage because there are relatively few side effects, and the use of zinc salts prevents the development of its deficit [131].

## Conclusion

**Fig. 3:**
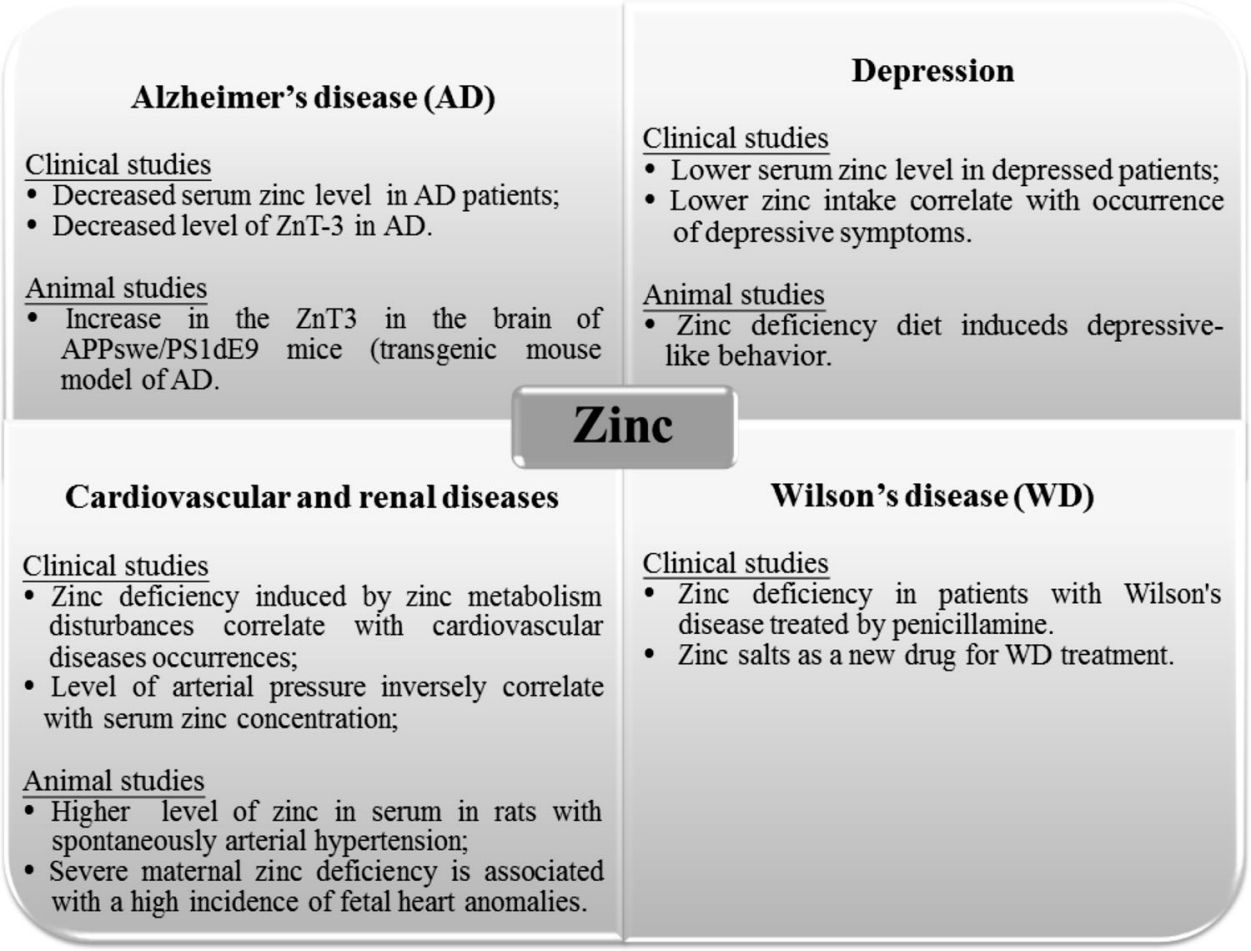
The possible role of the excess and efficiency of the zinc in different diseases

This review of the literature shows that zinc deficiency is connected with many diseases. Research related to the deficit of this element has been performed for over 50 years, but there are still no satisfactory theories that can be integrated with the many effects of zinc deficiency. The authors hope that the article represents a review of current knowledge in a clear and innovative way about the mechanisms associated with zinc deficiency in many diseases and metabolic disorders (Fig. 3). The summarized effects of zinc deficiency in different parts of the body are presented in Table 1.

**Table 1:** Effects of the zinc deficiency in different part of body

The organ	Effects of zinc deficiency	Source
Brain	Neuropsychiatric disorders	[17, 23, 59, 61, 132, 133]
	Neurosensory disorders	[16, 60, 132, 134, 137]
	Decreased nerve conduction	[132, 135]
	Mental lethargy	[14, 16, 17, 136]
Thymus	Thymic atrophy	[4, 63, 64, 69, 71]
Skin	Skin lesions	[9, 10, 16, 20, 136]
	Acrodermatitis	[13, 16, 17, 28, 31, 34]
	Decreased wound healing	[16–18]
Reproductive system	Hypogonadism	[16, 17, 33]
